# Association of early life factors and brain tumour risk in a cohort study

**DOI:** 10.1038/sj.bjc.6604615

**Published:** 2008-08-19

**Authors:** M M Cantwell, M R Forman, R J Middleton, L J Murray

**Affiliations:** 1 Centre for Clinical and Population Sciences, Queen's University Belfast, Mulhouse Building, Grosvenor Road, Belfast BT12 6BJ, UK; 2 Cancer Prevention Fellowship Program, National Cancer Institute, National Institutes of Health, Bethesda, MD, USA; 3 Department of Epidemiology, UT MD Anderson Cancer Center, PO Box 301439, Houston, Texas 77030-3721, USA; 4 Northern Ireland Cancer Registry, Centre for Clinical and Population Sciences, Queen's University Belfast, Mulhouse Building, Grosvenor Road, Belfast BT12 6BJ, UK

**Keywords:** brain tumour, childhood, early life factors

## Abstract

Using population-based linked birth and cancer registry data, we investigated whether the risk of brain tumour in childhood (*n*=155) was associated with perinatal risk factors. This population-based cohort showed that being born into a larger family or to a mother with a history of miscarriage may increase childhood brain tumour risk.

An increase in the incidence of central nervous system tumours has been observed during recent decades in a number of countries (Parkin *et al*, 1998), which could be as a result of improvements in diagnostic practise or because of changes in environmental exposures. The only consistent risk factor for brain tumour is the genetic syndrome the Li–Fraumeni syndrome (Narod *et al*, 1991).

Risk factors for cancers that occur during childhood and early adulthood are likely to differ from those in older people, with important influences operating during the perinatal and childhood period. There has been a growing interest in such relationships for risk of brain tumour, although the results of studies are largely inconsistent (Von Behren and Reynolds, 2003).

We have investigated the association between perinatal factors collected in a population-based cohort study in Northern Ireland, including birth weight, gestational age, the number of previous miscarriages, mother's and father's year of birth, social class, method of infant feeding, and household density at the time of the birth and early brain tumour risk, that is at age 1–30 years.

## Materials and methods

Since 1971, the Northern Ireland Child Health System has collected information on all births to mothers' resident in Northern Ireland. The Department of Health, Social Services and Public Safety maintains the data relating to births between 1971 and 1986. Subject to an appropriate confidentiality agreement, access was granted to these data that include date and place of birth; birth weight (in grams); gestational age (to the nearest complete week); the number of previous pregnancies, live births, and miscarriages; mother's and father's year of birth; social class (based on father's occupation); method of infant feeding on discharge from the place of confinement; the number of persons aged above and below 15 years per household at the time of birth of the index child; and the number of living rooms and bedrooms in the household at the time of the birth. Maternal age at delivery, birth weight, and gestational age were taken directly from birth notification forms completed in obstetric units. The other information was collected by health visitors in the home typically within 1–2 weeks of birth. Details of incident cases of brain tumour in Northern Ireland between 1975 and 1997 were obtained from the Northern Ireland Cancer Registry. Using sex, surname, and date of birth, cases born in Northern Ireland between 1971 and 1986 were identified within the Child Health System database.

### Statistical analyses

As we did not actively follow-up the cohort for migration or death, we did not feel justified in calculating person-years of risk and modelling the risk of disease using Poisson or Cox regression. Instead, we approximated relative risks by calculating the odds ratios obtained from unconditional logistic regression models, an approximation that is reasonable for a rare disease such as brain tumour. Perinatal information about the children who subsequently developed a brain tumour was compared with children who did not develop a brain tumour using a *χ*^2^ test. All statistical analyses were performed using SAS® (version 8.2) and two-sided *P*-values are reported. Ethical approval was obtained from the Research Ethics Committee of the Queen's University of Belfast.

## Results

The Child Health System contained data on 444 168 live births delivered between January 1971 and December 1986 in Northern Ireland. The Registrar General's Office, which registers all births within Northern Ireland, registered 444 111 live births for the same period. Only live births were included in this analysis. Newborns with congenital malformations (8883), missing birth weight or gestation age (4923), who were part of a twin or multiple gestations (*n*=9235), or with Down's syndrome (*n*=440) were excluded. The Northern Ireland Cancer Registry contains 251 patients diagnosed with brain tumours in Northern Ireland who were born between 1971 and 1986; of whom, 208 (82.9%) were identified within the Child Health System; 155 cases were malignant. The characteristics of the identified cases (*n*=155) and non-cases (*n*=420 436) are shown in Table 1. Mean age at diagnosis was 13.7 years (s.d. 7.61).

Table 1 shows the association between exposures of interest and risk of brain tumour. Boys had an increased risk of brain tumour as compared with girls, and children born between 1977 and 1978 were at an increased risk, although this may reflect differences in the completeness of data for earlier periods. Women who had a history of miscarriage were more likely to have a child with a brain tumour (OR=1.68; 95% CI: 1.16–2.42). Children who were born into households with ⩾3 children had a significantly increased risk of developing a brain tumour. There was no association between other perinatal factors and brain tumour risk.

Table 2 shows the mutually adjusted odds ratios for predictor variables and brain tumour risk. Newborn boys and newborns to mothers with a miscarriage history continued to be at an increased risk in the multivariate models. In addition, having either ⩾3 children or ⩾3 adults in the household at the time of the index birth increased the risk of brain tumour. Other perinatal factors were not associated with brain tumour risk in the multivariate model.

## Discussion

This study indicates that a greater number of children (⩾3) and adults (⩾3) in the house at the time of delivery were associated with increased brain cancer risk.

### Sex

Boys more than girls were at an increased risk of brain tumours, a finding that has been previously reported for both lymphomas and leukaemias (Rickert and Paulus, 2001; Baldwin and Preston-Martin, 2004); the underlying mechanism is not understood.

### Household density

The positive association between the number of children and adults in the household and brain cancer risk suggests that earlier exposure to infections in childhood may increase risk, perhaps due to immune modulation. In our study, there was no association between infant feeding practise and brain tumour risk. However, a recent study of brain tumour risk in relation to various indicators of infection during gestation and childhood highlighted two possible contradictory theories (Shaw *et al*, 2006). First, brain cancer risk was moderately reduced among those who were breastfed or attended daycare for more than 1 year, suggesting their immune system was strengthened by these surrogate markers of infection exposure. Secondly, brain cancer risk was positively associated with both direct (infection during gestation or childhood) and indirect indicators (having older siblings) of infection in their study in agreement with our results. This would suggest that exposure to an infective agent induced an abnormal immune response or was carcinogenic in nature.

### Birth weight

There was no clear association between birth weight and brain tumour risk, although the results suggest that large (>4 kg) babies were at an increased risk as compared with those of normal weight. To date, findings on birth weight and brain tumour risk have been inconsistent, although an association has been shown for high birth weight and astrocytoma risk (Emerson *et al*, 1991; Linet *et al*, 1996). The association between high birth weight and brain tumour risk is believed to be due to heavier babies having a greater number of cells, which increases their vulnerability to environmental carcinogenic exposures, or because they have larger organ size and concomitant altered metabolic pathways (Gold *et al*, 1979).

No consistent association has been shown in previous studies of maternal age and childhood brain cancer, although our study is in agreement with two recent case–control studies as well as a prospective study that reported negative results, thereby indicating the lack of an effect of an older maternal age at the time of delivery on childhood brain tumour risk (Linet *et al*, 1996; Heuch *et al*, 1998; Schuz *et al*, 2001). Paternal age was not associated with risk of brain tumours in this study.

A possible association with prior foetal loss has been investigated in numerous case–control studies, which have shown conflicting and inconsistent risk estimates perhaps due to selective participation and recall bias (Von Behren and Reynolds, 2003). Our cohort study showed an increased risk for newborns to women with a miscarriage history, and as our findings have minimal biases, they may be more close to the true relationship. To our knowledge, this is the first cohort study to link miscarriage history with brain tumour risk in the offspring.

A major strength of this study is the follow-up of a whole population for virtually the full period of risk for childhood brain tumour. Case ascertainment was high as patients are diagnosed and treated in a few centres within a relatively small geographic region. Failure to identify birth records of cases born during 1971–1986 was likely to be entirely random, with the exception of children moving into Northern Ireland, which was uncommon. These data in the analyses were documented before the onset of the illness, thus avoiding recall bias and observer bias and as the Northern Irish population is ethnically homogenous, racial (and genetic) factors may be largely discounted.

The study has some potential limitations. The relatively small number of cases precludes potentially important subgroup analyses. Data were collected by many observers and, although attempts were made to standardise procedures, variation in recording is likely to have occurred. However, such misclassification bias will result in an attenuation of real associations. The matching process will not have identified brain tumour cases born outside marriage, if they had a subsequent name change. As unmarried mothers are more likely to be younger and live with relatives (higher household densities) than their married counterparts, failure to identify these cases may have reduced the power of the study to detect associations between household densities, or young maternal age, and brain tumour risk. However, as birth records were identified for almost 83% of possible cases, this is not a major issue.

This population-based study is the first cohort study to demonstrate that being born to a mother with a history of miscarriage or into a larger family may be a risk factor for brain tumours. Further investigation of this hypothesis is warranted, including the examination of infections that are likely to be transmitted placentally *in utero* and during early childhood.

## Figures and Tables

**Table 1:** Association between selected early life factors and birth characteristics and risk of brain tumour, univariable analyses

**Variable**	**Cases *N* (%)**	**Non-case *N* (%)**	**Odds ratio (95% CI)**	***P*-value/trend**
*Sex*				
Male	99 (63.9)	216 535 (51.5)	**1.67** **(1.20, 2.31)**	
Female	56 (36.1)	203 901 (48.5)	1.0 (ref)	0.002
				
*Year of birth*				
1971–1972	16 (10.3)	57 562 (13.7)	1.0 (ref)	
1973–1974	12 (7.7)	51 299 (12.2)	0.84 (0.40, 1.78)	
1975–1976	24 (15.5)	49 685 (11.8)	1.74 (0.93, 3.27)	
1977–1978	33 (21.3)	49 920 (11.9)	**2.38 (1.31, 4.32)**	0.001
1979–1980	20 (12.9)	53 793 (12.8)	1.34 (0.69, 2.58)	
1981–1982	17 (11.0)	52 212 (12.4)	1.17 (0.59, 2.32)	
1983–1984	13 (8.4)	52 680 (12.5)	0.89 (0.43, 1.85)	
1985–1986	20 (12.9)	53 283 (12.7)	1.35 (0.70, 2.61)	0.007/0.95
				
*Maternal age (years)*				
<25	48 (31.0)	155 625 (37.1)	0.76 (0.53, 1.08)	
25–34	88 (56.8)	217 232 (51.8)	1.0 (ref)	
⩾35	19 (12.3)	47 447 (11.1)	1.01 (0.62, 1.67)	0.29/0.16
				
*Paternal age (years)*				
<25	33 (23.2)	85 939 (22.8)	1.14 (0.75, 1.72)	
25–34	73 (51.4)	216 059 (57.4)	1.0 (ref)	
⩾35	36 (25.4)	74 160 (19.7)	1.44 (0.96, 2.14)	0.20/0.34
				
*Social class*				
Non-manual	39 (25.2)	89 469 (21.3)	1.24 (0.87–1.79)	0.24
Manual/other	116 (74.8)	330 967 (78.7)	1.0 (ref)	
				
*Miscarriage history*				
None	99 (71.2)	308 778 (80.6)	1.0 (ref)	
⩾1	40 (28.8)	74 486 (19.4)	**1.68 (1.16, 2.42)**	0.005
				
*Gestation age (weeks)*				
<38	9 (5.8)	34 522 (8.2)	0.68 (0.34, 1.39)	
38–39	52 (33.6)	136 390 (32.4)	1.0 (ref)	
⩾40	94 (60.7)	249 524 (59.4)	0.99 (0.70, 1.39)	0.55/0.47
				
*Presumptive birth order*				
First born	41 (28.5)	115 578 (29.7)	1.0 (ref)	
Not first born	103 (71.5)	274 095 (70.3)	1.06 (0.74, 1.52	0.75
				
*Breast fed*				
Yes	29 (18.7)	77 969 (18.5)	1.0 (ref)	0.76
No	120 (77.4)	320 573 (76.3)	1.01 (0.67–1.51)	
				
*Birth weight (g)*				
<2500	5 (3.2)	19 218 (4.6)	0.73 (0.30, 1.79)	0.18/0.07
2500–4000	127 (81.9)	356 887 (84.9)	1.0 (ref)	
>4000	23 (14.8)	44 331 (10.5)	1.46 (0.91, 2.27)	
				
*Number of children in household* <*15 years*^a^				
<3	44 (71.0)	143 217 (82.1)	1.0 (ref)	
⩾3	18 (29.0)	31 160 (17.9)	**1.88 (1.09, 3.25)**	0.02
				
*Number of adults in household*				
<3	73 (87.0)	214 582 (92.4)	1.0 (ref)	
⩾3	11 (13.1)	17 576 (7.6)	1.84 (0.98, 3.47)	0.06
				
*Household density*				
<1 person/room	11 (7.1)	37 272 (8.9)	1.0 (ref)	0.74
⩾1 person/room	51 (32.9)	136 580 (32.5)	1.27 (0.66–2.43)	

a Includes the newborn.

**Table 2:** Mutually adjusted relative risks for the association between predictor variables and brain tumour risk

**Variable**	**Categories**	**Odds ratio (95% CI)**	***P*-value**
Sex^a^	Male	**1.68 (1.21, 2.33)**	0.003
	Female	1.0 (ref)	
			
Maternal age (years)^a^	<25	0.70 (0.44, 1.12)	
	25–34	1.0 (ref)	0.86
	⩾35	0.79 (0.45, 1.40)	
			
Paternal age^a^	<25	1.49 (0.89, 2.50)	
	25–34	1.0 (ref)	0.35
	⩾35	1.43 (0.90, 2.29)	
			
Social class^a^	Non-manual	1.19 (0.82, 1.75)	
	Manual/other	1.0 (ref)	
			
Miscarriage history^a^	None	1.0 (ref)	
	⩾1	**1.64 (1.13, 2.40)**	0.01
			
Gestation age (weeks)^a^	<38	0.70 (0.34, 1.42)	
	38–39	1.0 (ref)	0.36
	⩾40	1.03 (0.73, 1.44)	
			
Presumptive birth order^a^	First born	1.0 (ref)	
	Not first born	0.90 (0.60, 1.34)	
			
Breast fed^a^	Yes	1.0 (ref)	
	No	1.09 (0.71, 1.66)	
			
Birth weight (g)^a^	<2500	0.63 (0.30–1.312)	0.26
	2500–4000	1.0 (ref)	
	>4000	1.27 (0.84, 1.92)	
			
Number of children in household <15 years^b^,^c^	<3	1.0 (ref)	
	⩾3	**2.20 (1.12, 4.32)**	0.02
			
Number of adults in household^c^	<3	1.0 (ref)	
	⩾3	**2.93 (1.33, 6.49)**	0.008
			
Household density^c^	<1 person per room	1.0 (ref)	
	⩾1 person per room	1.01 (0.40, 2.55)	

a The analyses are based on 124 cases (80%) and 332 650 non-cases (79.1%) who had information available for all variables except number of children and adults in household and household density. Adjusted for year of birth (categorical) sex, maternal age, paternal age, social class, miscarriage history, gestation age, birth order, breastfed, and birth weight as appropriate.

b Includes the newborn.

c The analyses are based on 52 cases (33.5%) and 145 364 non-cases (34.6) who had information available on all variables. Adjusted for year of birth, sex, maternal age, paternal age, social class, miscarriage history, gestation age, birth order, breastfed, birth weight, number of children in household, numbers adults in household, and household density as appropriate.
